# The Correlation between Aquaporin-4 Antibody and the Visual Function of Patients with Demyelinating Optic Neuritis at Onset

**DOI:** 10.1155/2015/672931

**Published:** 2015-05-11

**Authors:** Hui Yang, Wei Qiu, Xiujuan Zhao, Wei Xiao, Shaofen Lin, Yan Luo, Lin Lu

**Affiliations:** ^1^State Key Laboratory of Ophthalmology, Zhongshan Ophthalmic Center, Sun Yat-sen University, Guangzhou 510060, China; ^2^Department of Neurology, the Third Affiliated Hospital of Sun Yat-sen University, Guangzhou 510630, China

## Abstract

ON patients with AQP4-Ab seropositivity tend to be predominantly female and young and have worse visual acuity and more severe damage to their visual fields compared with AQP4-Ab seronegativity.

## 1. Introduction

Optic neuritis (ON) is an acute demyelinating disease of the optic nerve that can destroy visual acuity, altering it from normal to full blindness within a few days. The severity and prognosis of ON vary enormously because of different etiologies. The etiology of ON remains elusive in many cases [1]. ON may be the first symptom of neuromyelitis optica (NMO), multiple sclerosis (MS), or another autoimmune disease, such as systemic lupus erythematosus (SLE). When the etiology cannot be found at the moment, these patients are diagnosed as idiopathic demyelinating ON. But this does not mean they will not develop NMO or MS or show signs of another autoimmune disease at a later stage.

It is extremely important to determine the etiology behind each ON patient because the etiology determines the treatment, prevention, and prognosis of each patient. There are many ways to determine the etiology of ON. Cerebrospinal fluid (CSF) examination and magnetic resonance imaging (MRI) are helpful to identify MS-related ON, while a serum immunological exam is used to identify autoimmune-disease-related ON. But it had been extremely difficult to distinguish NMO-related ON from other types of ON.

While the AQP4 antibody (AQP4-Ab) was found to be specifically expressed in the NMO in 2005 [2], it had been considered to be pathogenic to NMO later on [3]. AQP4-Ab seropositive status has important prognostic and therapeutic implications for patients with NMO-related ON. ON patients with AQP4-Ab seropositivity were classified as belonging to the NMO disease spectrum (NMODS) in 2007 [4], which means these patients are actually in a stage of NMO and more likely to convert to typical NMO. When an ON patient is considered to be more likely to convert to NMO, the treatment will accordingly be different. So, it is very important and necessary to find out the AQP4-Ab status of patients with ON in order to diagnose NMO-related ON as soon as possible.

The prevalence of AQP4 antibody in ON among Caucasians is quite low [5–8], which is consistent with the fact that Caucasian ON patients are more likely to convert to MS than NMO [9]. However, the prevalence of AQP4-Ab in ON among Asians, who are more likely to convert to NMO, is not very clear. The reported AQP4 seropositive rates in Asian ON patients from different study groups vary a lot [10–15], but all are higher than that of Caucasian ON patients. So, the utility of the AQP4-Ab test for Asian ON patients needs to be further investigated.

It has been reported that ON patients with NMO have the characteristic of severe visual damage upon attack and a poor prognosis [15]. But the correlation between AQP4-Ab and damage to the visual function of ON patients at onset has not been studied yet. Therefore, AQP4-Ab was tested in consecutive ON patients at Zhongshan Ophthalmic Center in this study, and the correlation between AQP4 seropositive status and visual function damage at onset was analyzed to evaluate the value of AQP4-Ab testing for Chinese ON patients.

## 2. Patients and Method

This was a cross-sectional, single-center, case-control study. After the protocol was approved by the Institutional Review Board of Zhongshan Ophthalmic Center, Sun Yat-sen University, Guangzhou, China, patients gave written informed consent to participate in this study. The study cohort consisted of patients who were diagnosed as acute ON at Zhongshan Ophthalmic Center from November 2013 to March 2014. The diagnostic criteria were as follows: an acute visual acuity decrease with or without eye pain; an optic nerve tract pattern of visual field (VF) damage; and the appearance of at least one of the following signs: a relative afferent pupillary defect (RAPD) or a visual evoked potential (VEP) abnormality. ON patients were excluded if they showed any clinical or laboratory evidence of compressive, ischemic, toxic, hereditary, metabolic, or infusive optic neuropathy or other ocular or nerve system diseases that can cause acute optic neuropathy. The ON patients who were found to have systemic diseases at the beginning were also excluded from this study.

A recurrent ON (RON) case was defined as follows: at least two episodes of optic neuritis attacks with an interval between the attacks of ≥4 weeks that was not due to a sudden stop or decrease of corticosteroid. Otherwise, the ON was defined as an isolated ON (ION). The diagnosis of ION or RON was made based on medical history. The follow-up time ranged from 0.2 to 4.8 months (2.44 ± 1.47 months).

All patients with complete medical histories underwent routine neurological examinations, brain MRIs, and ophthalmological examinations, including best corrected visual acuity (BCVA), intraocular pressure, slit lamp and fundus examination, VF and VEP, laboratory testing, including blood routine, HIV HBV HCV Syphilis, mitochondrial DNA sequencing, and a profile of autoantibodies, including antinuclear antibody (ANA), extractable nuclear antigen antibodies (SSA/SSB), rheumatoid factor (RF), anticardiolipin antibodies (ACA), and antithyroglobulin antibody (ATG) [16].

Blood was obtained before the corticosteroid therapy. AQP4-Ab was tested at the Third Affiliated Hospital of Sun Yat-sen University, Guangzhou, China. The AQP4-Ab was detected by the indirect immunofluorescence using human AQP4-transfected cells from a commercial BIOCHIP kit (Euroimmun, Germany). The cutoff value for positivity (minimal dilution of serum) was 1 : 4. Clinical data were recorded together with the AQP4-Ab. Patients were subdivided into an AQP4 seropositive group and an AQP4 seronegative group, according to the results of the AQP4-Ab test.

### 2.1. Statistical Analysis

All data are expressed as mean ± SD. Statistical analysis was performed using STATA 12.0 software (Stata Corporation, College Station, TX, USA). A two-group independent *t*-test was used to compare quantitative material. A Chi-square test was used to analyze the grouped data. A value of *p* < 0.05 was considered statistically significant.

## 3. Results

### 3.1. Seroprevalence of AQP4-Ab in Patients with ON

Screening for serum AQP4-Ab was conducted in 36 acute ON patients (29 women and 7 men), among which 19 patients (52.8%) were anti-AQP4-Ab positive and 17 patients (47.2%) were anti-AQP4-Ab negative. The seropositive rate for AQP4-Ab was significantly higher in patients with RON than ION (9/13, 69.2% versus 10/23, 43.5%; *p* < 0.05). The characteristics of patients with ON were presented in Table 1. The female/male ratio was considerably higher in the seropositive group than the seronegative group. The mean age at onset of all patients was 37.3 ± 16.53 y. Patients in the AQP4 seropositive group were younger than those in the AQP4 seronegative group. As for the mean age of the first eye attack, it was much lower in the AQP4 seropositive group than AQP4 seronegative group.

None of these patients showed ON-related signs of brain lesions on their MRIs. Leber's hereditary optic neuropathy (LHON) was excluded due to the negative results of all the tests of mitochondrial DNA sequencing. None of autoantibody test results met the diagnostic criteria for Sjögren's syndrome, SLE, or other autoimmune diseases (Table 1).

### 3.2. Accompanied Symptoms

No patients had spinal symptoms in the AQP4 seronegative group at onset and the six-month follow-up, while there were six patients in the seropositive group showing spinal symptoms during the six-month follow-up (*p* < 0.001). No significant differences in the rate of prodrome and eye pain were found between the two groups.

### 3.3. Visual Function Findings

No significant differences were found between the two groups regarding the rate of binocular involvement (9/19 versus 10/17, *p* > 0.05). At the onset of ON, 25 of 27 (92.6%) attacked eyes had VA ≤ 0.05 in the seropositive group, while 10 of 27 (37.0%) attacked eyes had VA ≤ 0.05 in the seronegative group. The VA distribution mainly ranged from NLP to 0.05 in the seropositive group, while being >0.05 in the seronegative group (*p* < 0.001).

Due to poor VA, the eyes in the seropositive group that failed the VF exam totaled 19 of 31 (61.2%), while in the seronegative group that total was only 6 of 27 (22.2%) patients (*p* < 0.05). There was no significant difference in the pattern of VF damage between the two groups (*p* > 0.05), but the mean deviation of VF in the seropositive group was worse than that of the seronegative group (−23.89 ± 7.65 versus −10.85 ± 9.50; *p* < 0.01).

P100 of VEP was not elicited in 15 of 27 (55.6%) eyes at onset in the seropositive group and 7 of 27 (25.9%) eyes in the seronegative group, while P100 was delayed in 9 (33.3%) eyes in the seropositive group and 15 (55.6%) eyes in the seronegative group. Significantly more eyes with AQP4-Ab seropositivity showed a lack of the P100 component than seronegative patients (*p* < 0.05) (Table 2).

## 4. Discussion

Isolated ON (ION) patients with seropositive AQP4 antibody are considered to be involved in the NMO spectrum disorder (NMOSD) now and are more likely to develop definite NMO after a period of time [4]. AQP4-Ab is considered to be a pathogenetic and prognostic factor of NMO-related ON. The distinctive nature of the optic nerve lesion between AQP4-Ab seropositive and seronegative patients with ON has been reported [14]. Optic nerve lesions are supposed to be more necrotic in the AQP4-Ab seropositive patients with ON and more demyelinating in the AQP4-Ab seronegative patients with ON [17]. However, the prevalence of AQP4-Ab in acute ON is not clear in the Chinese population, and its relationship with the severity of visual function of ON patients at onset has not been fully studied yet.

In this study, there was a high seroprevalence for AQP4-Ab (52.8%) in a relatively large cohort of Chinese patients with ON, while this seroprevalence stood at 69.2% in recurrent ON and 43.5% in isolated ON. The seroprevalence was much higher than that reported in American and Europe cases (3–5%) [6–8] and another 13 Chinese ON cases [10]. The AQP4-Ab positive rate has been reported to be 20% (5/25) in patients with RON by the Mayo Clinic, USA [15]. However, the prevalence varies considerably among the Chinese population. A high seroprevalence for AQP4-Ab (32.4%) has been reported in a study of 34 Chinese patients with severe ON [12] but only totaled 13/190 (6.8%) in mixed ON (RON and ION) [13]. Takagi et al. [14] has reported that the 3 of 32 (9.4%) Japanese patients with seropositive AQP4-Ab are all binocular and female with severe visual acuity damage. As shown in Table 3, there are no significant differences in age and sex distribution among the studies. Since the reliability and facticity of positive AQP4-Ab have been reconfirmed, the following factors might be responsible for the high seroprevalence of AQP4-Ab in our study. First, there was an ethical bias. The rate of NMO in demyelinating disease is 1-2% in Caucasians, but as high as 20–48% in Asians [9]. Asian ON patients have been considered to be more closely related to NMO. Second, patient source bias was another very important and main factor. Zhongshan Ophthalmic Center is the largest eye center in China. Therefore, ON patients with more severe visual damage are more likely to be referred to this center by the local hospital or to self-refer there. Third, serum samples for the AQP4-Ab test were collected before the corticosteroid treatment, considering that corticosteroid and immune suppression treatment suppress the expression of AQP4-Ab in serum [18].

There are debates about the utility of the AQP4-Ab test in evaluating the prognosis of isolated acute ON due to the very low seropositivity (~5%) of AQP4-Ab in the West [8]. As a result, it has been recommended to be tested only in patients with bilateral ON, recurrent ON, poor visual recovery, ON associated with immune disease, and an atypical MRI change of MS [19]. However, high seropositivity in our study and other studies [10] in China suggest the value of a routine AQP4-Ab test at the onset of ON in Chinese patients and perhaps other Asian patients, especially those with very severe visual damage at onset. AQP4-Ab has been proven to be the specific antibody of NMO. Matiello et al. have reported that 50% of AQP4-Ab seropositive patients with recurrent ON [15] progress to NMO within about 8.9 years. The AQP4-Ab test helps patients to obtain an early diagnosis and proper treatment. Since ON patients with NMO have a poor prognosis and require aggressive immunotherapy, identifying NMODS patients will enable the doctors to develop a suitable therapy strategy, for example, using a low dose of corticosteroid and immunosuppressant to prevent a relapse. What is more important is that harmful treatments for NMO, such as interferon [20], natalizumab [21], and fingolimod [22], can be avoided.

Our study showed a marked female predominance, which was consistent with another report [23]. The mean age of both the acute attack and first eye attack was lower in the AQP4-Ab seropositive group. It has been reported that about one-half of NMO patients present with isolated ON [24, 25] and the ON in NMO is characterized by profound and persistent visual function damage. Matiello et al. [15] and Lai et al. [12] found worse visual acuity and poorer prognoses in the AQP4-Ab seropositive patients. In our study, besides visual acuity, VF and VEP were also tested to fully represent the visual function. We found that worse visual acuity and visual field were more common in seropositive patients; the mean deviation of VF in the seropositive group was worse than that of the seronegative group; and more seropositive patients showed a lack of VEP responses than seronegative patients. These results confirmed that seropositive patients with ON had more severe visual function damage. Therefore, the close correlation between AQP4-Ab and visual function damage at onset was confirmed in this cross-sectional study.

There were some limitations in this study: First, no definite causal conclusions can be made with a cross-sectional design and without the follow-up results. Second, the sample size was small. Last, more indexes of visual function could be observed, such as color vision and contract sensitivity, to further investigate the AQP4 antibody's effect on visual function damage. A prospective, multicenter, longitudinal cohort study of patients with ON needs to be further investigated in the future.

In conclusion, AQP4-Ab seropositive ON patients tend to be predominantly female and younger, with worse visual acuity and more severe damage to their visual fields. AQP4-Ab seropositivity indicates patients with ON might have worse visual function and prognoses.

## Figures and Tables

**Table 1:** Characteristics of patients with ON.

	AQP4-Ab seropositive (*n* = 19)	AQP4-Ab seronegative (*n* = 17)	*p*
Sex, F : M	18 : 1	11 : 6	0.023^∗∗^
Age at onset, y (mean ± SD)	32.68 ± 15.75	42.53 ± 16.26	0.037^∗^
Age at first eye attack, y (mean ± SD)	30.84 ± 15.71	41.06 ± 16.11	0.031^∗^
Patients with multiple episodes of ON (more than once), number	9	4	0.137^∗∗^
ANA, number	2	1	—
Anti-dsDNA, number	1	0	—
ANCA, number	1	1	—
Anti-SSA, number	1	0	—
Anti-SSB, number	1	0	—
ATG, number	1	0	—
RF, number	0	0	—

^∗^ *t*-test, ^∗∗^Chi-square test.

**Table 2:** Visual function of patients with ON.

	AQP4-Ab seropositive	AQP4-Ab seronegative	*p*
Eyes of visual acuity, number			
NLP	6	2	<0.001^∗∗^
LP-0.05	19	8	
>0.05	2	17	
Eyes of visual evoked potential, number			
Unevoked	15	7	0.04^∗∗^
Delayed	9	15	
Normal	3	5	
Eyes failed VF exam, number	19	6	<0.001^∗∗^
Visual field MD (mean ± SD)	−23.89 ± 7.65	−10.85 ± 9.5	<0.001^∗^

^∗^ *t*-test, ^∗∗^Chi-square test.

**Table 3:** Frequency of AQP4-Ab in ON among different countries.

	Ethnicity	Age	M : F	AQP4-Ab seropositive		
				Total (%)	RON (%)	ION (%)
Our study	China, South	37.3	7 : 29	19/36 (52.8)	8/13 (69.2)	10/23 (43.5)
Long et al. [10]	China, South	31.25	4 : 9	4/13 (30.8)	3/8 (38)	1/5 (20)
Chan et al. [11]	China, South	—	—	3/23 (13)	2/9 (33)	1/14 (14)
Lai et al. [12]^∗^	China, North	31.1	12 : 22	11/34 (32.4)	5/11 (45.5)	6/23 (26.1)
Li et al. [13]	China, North	38.89	55 : 135	13/190 (6.8)	10/79 (12.7)	3/114 (2.6)
Takagi et al. [14]	Japan	46	13 : 19	3/32 (9.3)	—	—
Matiello et al. [15]	American	—	2 : 23	—	5/25 (20)	—
Banwell et al. [5]^∗∗^	^#^Multi	12.6 (7.4–17.9)	1 : 1.2	1/13 (7.7)	1/5 (20)	0/8 (0)
Petzold et al. [6]	UK	29	3 : 6	4/77 (5.2)	2/36 (6)	2/41 (5)
Costa et al. [7]	Spain	28 (13–48)	34 : 67	3/101 (3.1)	—	—
Jarius et al. [8]	^##^Multicountries	34	35 : 104	8/139 (5.8)	5/50 (10)	3/89 (3.4)

ION: isolated monophasic optic neuritis, RON: recurrent optic neuritis; ^∗^severe ON visual acuity of 20/200 or worse in at least 1 eye at the nadir; ^∗∗^pediatric cases; ^#^serum from multiple ethnicity of White, Asian, Black, Hispanic, Mestizo, Middle Eastern, and Native Argentinean; ^##^serum from Austria, Denmark, France, Germany, Italy, and Turkey.
